# Protective Effects of Hydrogen-rich Water Intake on Renal Injury in Neonatal Rats with High Oxygen Loading

**DOI:** 10.14789/jmj.JMJ21-0048-OA

**Published:** 2022-05-27

**Authors:** MASAHITO SAITOH, AMANE ENDO, AKINA MATSUDA, HIROKI MIYANO, YUSUKE GONDA, AKIRA MIZUTANI, TAICHI HARA, MAYU NAKAGAWA, KOJI SAKURAYA, YAYOI MURANO, NAOTO NISHIZAKI, SHUICHIRO FUJINAGA, YOSHIYUKI OHTOMO, TOSHIAKI SHIMIZU

**Affiliations:** 1 Department of Pediatrics and Adolescent Medicine, Juntendo University Graduate School of Medicine, Tokyo, Japan; 1 Department of Pediatrics and Adolescent Medicine, Juntendo University Graduate School of Medicine, Tokyo, Japan; 2 Department of Pediatrics, Juntendo University Urayasu Hospital, Chiba, Japan; 2 Department of Pediatrics, Juntendo University Urayasu Hospital, Chiba, Japan; 3 Division of Nephrology, Saitama Children's Medical Center, Saitama, Japan; 3 Division of Nephrology, Saitama Children's Medical Center, Saitama, Japan; 4 Department of Pediatrics, Juntendo University Nerima Hospital, Tokyo, Japan; 4 Department of Pediatrics, Juntendo University Nerima Hospital, Tokyo, Japan

**Keywords:** nephrogenesis, immature glomeruli, neonatal hyperoxia, molecular hydrogen

## Abstract

**Objectives:**

This study aimed to investigate the protective effects of hydrogen-rich water (HW) intake on renal injury in neonatal rats with high oxygen loading.

**Materials:**

We used pregnant and newborn Sprague-Dawley rats.

**Methods:**

Four groups were set up, with mother and newborn rats immediately after delivery as one group: RA-PW (room air and purified water), RA-HW (room air and HW), O_2_-PW (80% oxygen and purified water), and O_2_-HW (80% oxygen and HW). The newborn rats were maintained in either a normoxic (room air, 21% oxygen) or controlled hyperoxic (80% oxygen) environment from birth. Then, HW (O_2_-HW and RA-HW groups) or PW (O_2_-PW and RA-PW groups) was administered to parents of each group.

**Results:**

The number of immature glomeruli significantly increased in the O_2_-PW group (exposed to hyperoxia). Conversely, the O_2_-HW group had significantly fewer immature glomeruli than O_2_-PW group. In the RT-PCR analysis of kidney tissue, α-SMA, TGF-β, and TNF-α levels were significantly higher in the O_2_-PW group than in the RA-PW group and significantly lower in the O_2_-HW group than in the O_2_-PW group.

**Conclusions:**

HW intake can potentially reduce oxidative stress and prevent renal injury in neonates with high oxygen loading.

## Introduction

Advancement in perinatal care has improved the survival rate of premature and low-birth-weight infants, but the risk of developing organ dysfunction remains (Development Origins of Health and Disease [DOHaD] hypothesis)^1)^. Kidney injury (glomerular filtration rate decrease/proteinuria), hypertension, and renal failure reportedly occur in preterm and low-birth-weight infants^2)^. Oftentimes, preterm neonates are born while their renal system is still developing because normally, nephrogenesis is not completed until 34-36 gestational weeks^3)^. Thus, renal development continues after birth in these infants. However, the glomerular abnormalities and reduced glomerular formation, as well as increased proportion of immature glomeruli, are observed in this population. Thus, postnatal nephrogenesis may be potentially impaired^3, 4)^. Several children and adults born prematurely suffer from hypertension^5, 6)^, reduced kidney size^7, 8)^, and impaired renal function^9, 10)^, highlighting the long-term consequences of preterm birth on renal health. The cause of impaired renal development after preterm birth and the mechanisms through which it may predispose adults to renal disease are still largely unknown; exposure to oxygen in the extrauterine environment may be a contributing factor^11-13)^. At birth, infants are exposed to oxygen concentrations far exceeding the intrauterine levels^14)^, causing oxidative stress^15, 16)^, which they are particularly susceptible to because they have low antioxidant levels^17, 18)^. Macrophage infiltration, reactive oxygen species (ROS) activity, and renin-angiotensin system activation are essential in renal injury pathogenesis^19)^. Oxidative stress is a common pathway leading to chronic renal damage, with damage to cells, tissues, and organs caused by ROS^20)^. It has been implicated in numerous commonly occurring preterm birth-related conditions, including retinopathy of prematurity (ROP)^21)^. High oxygen load increases oxidative stress in rats, resulting in glomerular developmental disorders^22)^.

Alternatively, hydrogen reduces oxidative stress, effectively protecting the tissue from injury. Hydrogen selectively decreases the production of hydroxyl radical and peroxynitrite, which are two of the most cytotoxic ROS, and protects against oxidative stress^23)^. In rats, hydrogen-rich water (HW) and hydrogen saline significantly attenuate renal ischemia-reperfusion injury and reduce the serum levels of 8-OHdG (a biomarker of oxidative DNA damage)^24, 25)^. Several nephrology studies have also demonstrated the efficacy of hydrogen.

In this study, we aimed to investigate the effect of hydrogen on oxidative stress and glomerular developmental disorders in newborn rats with a high-concentration oxygen load.

## Materials and Methods

### Animals

The Juntendo University Animal Care Facility (Tokyo, Japan) approved all of our study procedures. Female Sprague-Dawley rats at gestational day 19 were purchased from Nihon SLC, Co., Ltd. (Shizuoka, Japan) and housed in individual cages in the same room at 24°C-25°C, with a relative humidity of 60% under a 12-hour/12-hour light/dark cycle and free access to food and water, at the Juntendo University Animal Care Facility. Pups were born naturally at term and maintained in either 80% oxygen (a mixture of medical-grade 100% oxygen and room air [RA]; Oxycycler ProOx 110; Biospherix, Lacona, NY, USA) or RA from postnatal day (PD) 0 to PD 12. The approval number obtained from the Juntendo University Animal Care Facility is 2020176.

### Experimental groups

Pregnant Sprague-Dawley rats were divided into four groups. From birth to PD 12 , two groups were bred in 80% oxygen, and the other two were in RA. HW (O_2_-HW and RA-HW groups) or purified water (PW; O_2_-PW and RA-PW groups) was administered to parents of each group after birth. To administer water and molecular hydrogen to newborn rats, there is a method of injecting water and molecular hydrogen directly into the stomach with a gastric catheter. However, this method had a problem in continuing the experiment for small rats. Therefore, with a view to future clinical application, we selected a method in which water and molecular hydrogen were orally administered to the mother rat and the effect was judged in the newborn rat raised by the mother rat’s breast milk. The pups were sacrificed at PD 19, and their kidneys were excised for tissue histologic examinations and biochemical assays. The weight of kidneys, number of glomeruli and immature glomeruli, and levels of other markers were compared and validated.

### Histopathological and immunohistochemical analyses

The harvested kidneys were fixed in 10% formalin, embedded in paraffin, and sliced into sections starting from the central region of the kidney across the full coronal plane. The slides containing the sliced sections were stained with hematoxylin and eosin and examined by optical microscopy. For each section, three microphotographs from the anterior, posterior, and mediolateral regions of the kidney, each including the full thickness of the cortex, were obtained using a digital camera (DS-L3; Nikon, Tokyo, Japan) at 400× magnification. To determine any changes in the number of nephrons, we counted the glomeruli and immature glomeruli in the whole section starting from the central region of the kidney across the full coronal plane in P19 pups. Mature glomeruli were defined as glomeruli with loose tuft structure, lobulation, and patent capillary loops lined with typical podocytes, whereas immature glomeruli referred to glomeruli with at least half of the circumference of capillary loops was densely lined with dark cuboidal epithelial cells. The lumen of the loops is generally narrow, with no tuft lobulation.

### Reverse transcription-polymerase chain reaction (RT-PCR)

The expression levels of α-SMA, TGF-β, and TNF-α in the renal cortex were determined by real-time RT-PCR using the TaqMan system according to the manufacturer’s protocol. The samples were homogenized with a buffer RLT added and rotated twice at 3000 rpm for 30 seconds using a rotor-stator homogenizer. TaqMan probe-based quantitative RT-PCR was conducted using cDNA synthesized from kidney biopsy. RNA was prepared using the High-Capacity cDNA Reverse Transcription Kit and analyzed using the default protocols of the 7500 Fast Real-Time PCR System (Life Technologies). Using the Standard Curve Method, we normalized each gene expression to GAPDH gene expression. Furthermore, primers and probes for α-SMA ACTA (Rn01759928_g1), TGF-β (Rn00572010_m1), and TNF-α (Rn99999017_ m1) were prepared using TaqMan Gene Expression Assays.

### Statistical analysis

Data are expressed as mean ± standard deviation. We used one-way analysis of variance to determine the differences between groups and the Bonferroni method for post hoc multiple comparisons. All statistical data were analyzed using SPSS Statistics software, version 17.0 (SPSS Inc., Chicago, IL, USA), and a P value of < 0.05 was considered statistically significant.

## Results

### Effect of hyperoxia on glomeruli

Hyperoxia exposure did not change the body weight, kidney weight, and the number of glomeruli per section. However, the number of immature glomeruli in the O_2_-PW group significantly increased (Figure 1a). The average number of immature glomeruli in the RA-PW and O_2_-PW groups was 14.8 ± 1.62 and 70.8 ± 3.30, respectively. Conversely, the O_2_-HW group (39.9 ± 2.38) had significantly fewer immature glomeruli (Figure 1b).

**Figure 1a:**
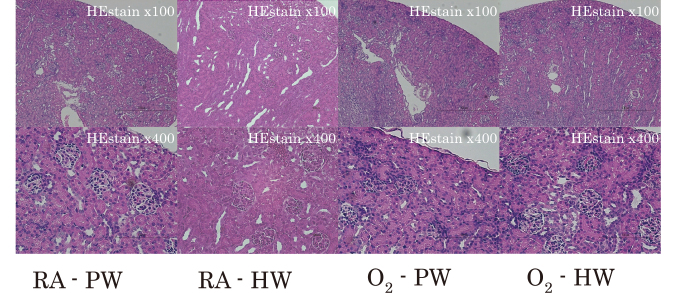
Histological images of the kidneys of newborn rats In the O_2_-PW group, the number of immature glomeruli significantly increased (Hematoxylin and eosin staining of 3μm-thick kidney sections; 100× and 400× magnification). Abbreviations: RA-PW, the group reared in room air with purified water; RA-HW, the group reared in room air with hydrogen-rich water; O_2_-PW, the group reared in 80% oxygen with purified water; O_2_-HW, the group reared in 80% oxygen with hydrogen-rich water.

**Figure 1b:**
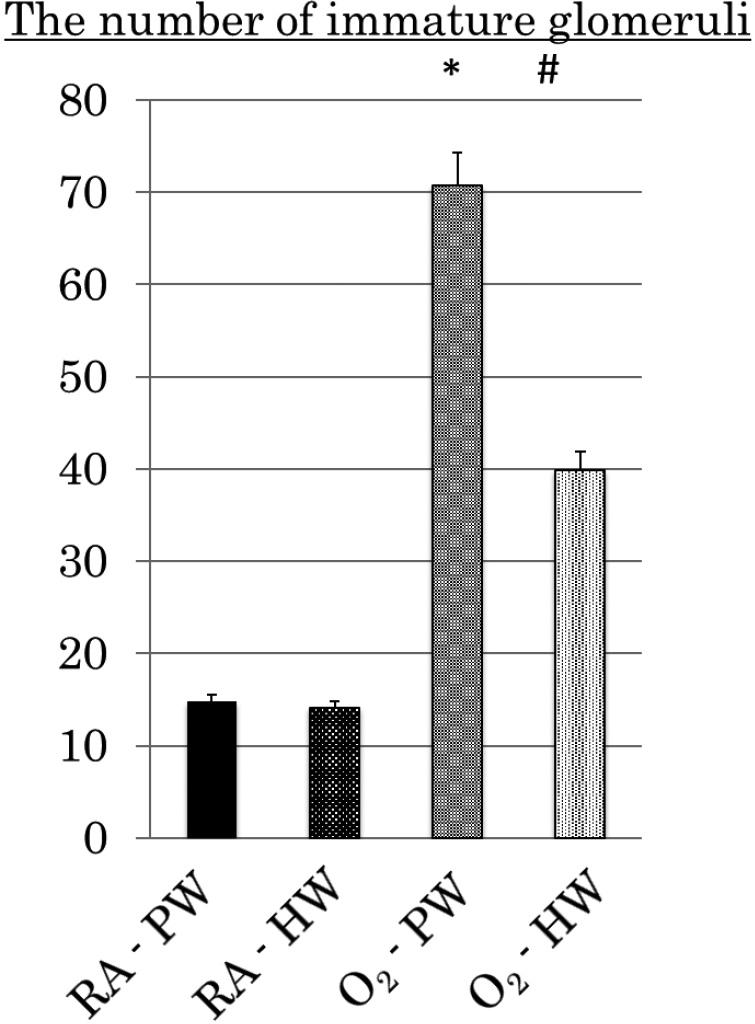
The number of immature glomeruli of newborn rats Hydrogen-rich water (HW) administration decreased the number of immature glomeruli.

### Effect of HW on α-SMA, TGF-β, and TNF-α mRNA expression in hyperoxic rats

**Figure 2:**
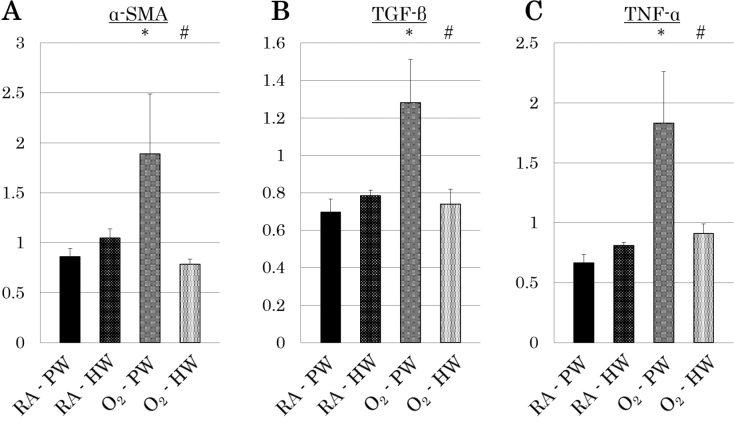
RT-PCR analysis of renal specimens In RT-PCR analysis, hyperoxia exposure led to the increase of α-SMA (A), TGF-β(B), and TNF-α(C) expression, and hydrogen-rich water (HW) administration decreased their expression.

The RT-PCR analysis of kidney tissue revealed characteristic findings in the O_2_-PW group. The O_2_- PW group had significantly higher α-SMA, TGF-β, and TNF-α levels than the RA-PW group (α-SMA: 1.89 ± 0.44 vs. 0.86 ± 0.07; P < 0.05; Figure 2-A) (TGF-β: 1.28 ± 0.24 vs. 0.70 ± 0.07; P < 0.05; Figure 2-B) (TNF-α: 1.83 ± 0.60 vs. 0.67 ± 0.08; P < 0.05; Figure 2-C). The O_2_-HW group also showed characteristic findings. The expression of α-SMA, TGF-β, and TNF-α was significantly suppressed in the O_2_-HW group than in the O_2_-PW group (α-SMA: 0.79 ± 0.08 vs. 1.89 ± 0.44; P < 0.05; Figure 2-A) (TGF-β: 0.74 ± 0.08 vs. 1.28 ± 0.24; P < 0.05; Figure 2-B) (TNF-α: 0.91 ± 0.05 vs. 1.83 ± 0.60; P < 0.05; Figure 2-C).

## Discussion

This study demonstrated that molecular hydrogen administration suppressed the generation of immature glomeruli caused by high-concentration oxygen administration. Yzydorczyk et al.^12)^ showed that nephron endowment was reduced by 25% in 25- to 35-week-old rats exposed to hyperoxia during postnatal nephrogenesis (80% oxygen from P3 to P10). Although the glomerular number decreased, they vaguely addressed whether hyperoxic exposure in the neonatal period directly impaired nephrogenesis because the end points were assessed in adult rats. Popescu et al.^26)^ reported that P5 rat pups exposed to hyperoxia showed a decreased glomerular size during postnatal nephrogenesis (80% oxygen from P3 to P10), whereas the P10 rat pups showed no change in glomerular count. Hyperoxia exposure in mice during postnatal nephrogenesis (65% oxygen from P0 to P7) did not cause overt adverse effects on renal development^27)^, but the glomeruli were enlarged in early adulthood. Nakagawa et al.^20)^ also reported that hyperoxia exposure suppresses glomerular development. Regarding the association between oxidative stress and immature glomeruli, the proportion of immature glomeruli increases in preterm birth^28)^. In our study, high oxygen levels significantly increased the number of immature glomeruli.

With regard to the relationship between oxidative stress and hydrogen, hydrogen selectively scavenges harmful ROS, such as hydroxyl radical and peroxynitrite; thus, nucleic acid oxidation and lipid peroxidation are mitigated, and the cells or tissues are protected against oxidant stress and apoptotic injury^23)^. Moreover, hydrogen increases the activity of antioxidant enzymes, including superoxide dismutase and catalase, and inhibits TNF-α and interleukin (IL)-6, thereby suppressing inflammation^29)^. In addition to directly neutralizing highly reactive oxidants, hydrogen indirectly reduces oxidative stress by regulating the expression of various genes. In the study of Nakashima et al., hydrogen decreased apoptosis and nephrotoxicity in rat models of cisplatin nephrotoxicity^30)^. Moreover, by reducing oxidative stress and suppressing the activation of inflammatory signaling pathways and cytokine production, HW prevented chronic allograft nephropathy in a kidney transplantation model; thus, the allograft function and overall survival improved^31)^. Therefore, HW can decrease oxidative stress and prevent damages in the kidneys.

Our study revealed that through hyperoxia exposure, the number of immature glomeruli can be significantly increased, but HW can significantly suppress such increase. The abovementioned studies suggest that molecular hydrogen suppresses active oxygen and is related to the suppression of renal development disorder. This study is the first to report that molecular hydrogen suppresses the production of immature glomeruli caused by high oxygen load.

In the RT-PCR analysis of kidney tissue, the levels of α-SMA, TGF-β, and TNF-α were significantly higher in the O_2_-PW group than in the RA-PW group. In contrast, they were significantly suppressed in the O_2_-HW group compared with those in the O_2_-PW group.

TGF-β induces fibrosis by promoting α-SMA expression and enhancing collagen fibronectin secretion. It also causes glomerular capillaries in rat renal development^32)^. Our study suggested that oxygen administration impaired the formation of glomerular capillaries and was suppressed by molecular hydrogen administration.

Angiotensin II elevation resulting from renal damage and oxidative stress promotes TNF-α production^33)^. The intravenous administration of TNF-α causes damage to glomerular endothelial cells and the glomerular endothelial surface layer^34)^. Our study suggested that TNF-α increased by oxidative stress damaged the glomerular endothelium but was suppressed by molecular hydrogen administration.

Most hydrophilic antioxidants cannot penetrate biomembranes and remain on the membrane surface, whereas molecular hydrogen administration can be distributed rapidly into lipids and cytosol and has no cytotoxicity, even at high concentrations^35)^. A study reported that inhalation of molecular hydrogen gas did not affect physiological parameters such as pH and blood electrolytes and had no adverse effects^36)^. In addition, oral HW intake is a useful route for molecular hydrogen administration because it is portable, safe, and does not alter the taste, smell, or pH of foods, drinks and drugs. In our study, no adverse events were observed in rats.

Our findings demonstrate the protective effect of HW intake on renal impairment. Therefore, HW may delay dialysis or kidney transplantation in children with renal impairment.

In conclusion, hyperoxia exposure during nephrogenesis causes renal impairment, whereas HW reduces oxidative stress and suppresses renal impairment.

## Funding

Juntendo University Project Research Funding (2019-42).

## Author Contributions

MS, AE, HM, AM, MN analyzed and interpreted the data regarding the newborn rats. AM, YG, TH, KS, YM, NN, SF, YO and TS prepared or revised for important intellectual content. AE was a major contributor in writing the manuscript. All authors read and approved the final manuscript.

## Conflicts of interest statement

The authors declare that there are no conflicts of interest.
